# Analogies Without Commonalities? Evidence of Re-representation via Relational Category Activation

**DOI:** 10.3389/fpsyg.2018.02441

**Published:** 2018-12-06

**Authors:** Nicolás Oberholzer, Máximo Trench, Kenneth J. Kurtz, Ricardo A. Minervino

**Affiliations:** ^1^Faculty of Psychology, University of Buenos Aires, Buenos Aires, Argentina; ^2^Department of Psychology, Universidad del Comahue and Instituto Patagónico de Estudios en Humanidades y Ciencias Sociales, Bariloche, Argentina; ^3^Department of Psychology, Binghamton University, Binghamton, NY, United States; ^4^Department of Psychology, Universidad del Comahue and Instituto Patagónico de Estudios en Humanidades y Ciencias Sociales, Cipolletti, Argentina

**Keywords:** analogy, mapping, re-representation, relational category, similarity

## Abstract

Analogies between cases with matching sets of connected relational structure is well-explained by existing theory. Re-representation is posited as an important mechanism to increase the flexibility of analogical processing by allowing the alignment of non-identical predicates across compared cases. It has been proposed that certain kind of categories can be characterized in terms of the relational structure that its exemplars tend to satisfy. Such relational categories have the property that all members of the category are analogous to one another. We ask whether a process of re-representation can alter the construal of a case and bring two evidently non-analogous cases into analogical alignment if they are both seen as members of the same relational category. We examine analogies between pairs of cases where the base is a canonical example of a relational category and the target would not be considered a member of the category on its own – critically, the cases themselves share no evident relational identities or similarities. In Experiment 1, we ask whether presenting a target case as part of an analogical pairing alters its construal. In Experiment 2, the pairs are presented for judgment as potential analogies. In both studies, participants interpret the target cases differently (consistent with the relational category) as a result of processing the analogy. There are two main implications: (1) a form of re-representation is at work in which the activation of a relational category triggers an alternate construal of the target case; and (2) this suggests a path to analogical status for cases that lack relational identities or similarities if the cases can both be fit to the same relational category.

## Introduction

In Argentine film “El fútbol o yo” (“Soccer or me”), Pedro Pintos is a fan of any the team or league – he watches soccer at home, at social gatherings and even at work. His passion for soccer dates from his childhood days and seemingly fits harmoniously with the rest of his life. However, his obsession starts to get out of control. Suddenly, Pedro finds himself separated from his wife and fired from his job due to his excessive commitment to watching soccer. Deep in misery, he finally realizes that his need for soccer is an *addiction* just like the abuse of alcohol. This insight leads him to look for help just as an alcoholic might do. This novel conceptualization of watching soccer as an addiction demonstrates a case of analogical re-representation. This re-representation serves as a precondition for Pedro matching his passion for soccer and his knowledge of alcohol dependency; and the analogical mapping provides a basis for further understanding and inference (e.g., the consequences of the addiction and how to deal with them). As this example illustrates, re-representation can allow unlikely matches and lead to novel interpretations that overcome previous ones (Gentner and Wolff, 2000; Kurtz, 2005).

Analogizing watching soccer to drinking alcohol requires accepting a similarity between two facts whose corresponding relations (represented by the verbs “watch” and “drink”) are fundamentally dissimilar actions that could only be considered similar at a ludicrously abstract level (“things people do”). Theoretical approaches to analogical re-representation like the widely held and broadly supported structure-mapping theory (Falkenhainer et al., 1989; Gentner, 1989), appear limited in their ability to explain analogies of this type. The difficulty arises from the requirement for some level of identical match between corresponding relations in order to accept two situations as analogous – on this view re-representation provides the basis for discovering or revealing such identical content while carrying out an alignment process between cases. In the present research, we propose an alternate path in which analogical re-representation takes place by transferring a relational category (e.g., *addiction*) that is readily activated by a base analog (e.g., abusive drinking of alcohol) but not readily activated by the target case (e.g., abusive watching of soccer). In other words, the soccer watching is highly unlikely to be seen as an example of addiction on its own, but when comparing soccer watching and drinking alcohol, an analogy is built not from any direct similarity between watching and drinking, but from the clear categorization of drinking alcohol as an example of addiction and the newly compelling re-representation of soccer watching as a viable member of the same relational category (*addiction*). We have carried out two experiments to evaluate the operation of a mechanism that operates on the meaning of propositions taken as wholes, so that the cognitive system identifies two situations as analogous on the basis of common relational category membership rather than finding an identity between dissimilar local relations.

### Re-representation in Structure-Mapping Theory

The structure-mapping theory of analogical reasoning states that forming an analogy consists of recognizing that two situations share a common structure of identical relations regardless of whether the corresponding entities are alike (Gentner, 1983, 1989, 2010; Gentner and Markman, 1997, 2006). In terms of propositional representations, relations are represented by multi-place predicates and entity attributes are represented by one-place predicates. Considering relatively simple cases, a situation like *John pushed the box of books* would be considered analogous to the situation *Mary pushed the wheelchair* because both are based on the same relation (an action represented by the verb *push*) while the entities filling the roles of agent (*John* vs. *Mary*) and object (*box of books* vs. *wheelchair*) differ. The theory is robust and predicts a wide range of psychological data. One facet of the explanatory framework that remains something of a frontier is re-representation (e.g., Day and Asmuth, 2017): the process of revealing semantic similarities between relations that are neither identical nor synonymous.

According to structure-mapping theory, two non-identical relations can be placed in correspondence either via minimal ascension (i.e., searching for their least abstract common superordinate; Falkenhainer, 1990) or semantic decomposition (i.e., breaking down the meaning of the paired relations into their semantic subcomponents; e.g., Yan et al., 2003). To continue the example above, *John pushed the box of books* could be considered analogous to the situation *Mary guided the wheelchair*, even though *push* is neither lexically identical nor synonymous to *guide*. In this case *push* and *guide* might be re-represented as two instances of causing directed change to an object’s position. Gentner and Kurtz (2006) found that semantically related verbs were commonly judged as analogous and required additional processing time – which was presumed to reflect the operation of a re-representation process.

### Re-representation Operating at the Level of Schema-Governed Categories

As noted, the structure-mapping theory requirement for alignment is that two relations are either identical or synonymous; or that re-representation operates to specify some form of direct semantic match. Along these lines, Gentner and Kurtz (2006) found a pattern of fast rejections (item pairs judged non-analogous) for verbs that were clearly unrelated. However, Minervino et al. (2008, 2013) have postulated that an element of similarity between relations may not always be necessary for two situations to be analogous: two situations with dissimilar relations can be considered analogous if they are instances of the same schema-governed relational category. Schema-governed categories (Markman and Stilwell, 2001; Gentner and Kurtz, 2005) are relational categories that specify the structure of situations or events in terms of a network of semantic interdependencies that hold among the constituents of the concept in question. As an example, the schema-governed category *physical aggression* tends to involve (a) an intentional agent, (b) a physical action exert by the agent, and (c) a patient sensitive to pain. This network not only includes several variables and constrains the types of entities or actions that may be bound to each of the variables in the schema, but it also stipulates how the values assigned to a variable constrain the values that can be bound to other variables. Following the example of physical aggression, if the object of the physical action is the *hair*, then a suitable action could be *to pull*; whereas if the target of the action is a *finger*, then a suitable action could be *to twist*. As these examples illustrate, various exemplars of a schema-governed category need not involve similar relations.

Minervino et al. (2008, 2013) studied sentence pairs such as *Sammy bought a perfume for a girl in his class* and *Sammy wrote a poem to a girl in his class*. The verbs are semantically distant and seemingly not a candidate for traditional re-representation, yet these pairs are usually judged to be analogous. One could imagine analogies across unrelated verbs as a result of idiomatic language (e.g., *hit the hay* vs. *go to sleep*), but the verbs used by Minervino et al. (2008, 2013) followed their literal meaning. The pairs were designed to include dissimilar verbs, but with both situations being members of the same relational category (in this case: *acts of romantic courtship*). As both cases activate the same schema-governed category, the events are seen as analogous despite the relations lacking similarity at the local level. Specifically, the *buying of perfume* and *writing of a poem* are both construed as situations in which (a) the agent intends to show generosity of spirit (b) the patient is capable of experiencing romantic emotions, and (c) the object is pleasant for the patient. The common semantic structure that derives from the interdependency between the verbs (*buy*, *write*) – which have no common basis between them – and the objects (*perfume*, *poem*) serves to activate a common schema-governed category and thereby drive analogical acceptance.

While comparisons of this kind do not require re-representing the analogs (each situation of the pair leads to the activation of the common schema-governed category independently from the other one), it is possible to envision cases where the target situation is not naturally represented as an instance of the schema-governed category to which the base situation belongs, but that might be re-represented as such due to taking part in analogical comparison with the base. Elaborating on the previous example, could a target situation like *Sammy played a joke to a girl in his class* become viewed as an act of romantic courtship through an analogical comparison with a base analog like *Sammy bought a perfume for a girl in his class*? The representational change in this case would involve assessing whether the target situation satisfies the semantic structure of the relational category activated by the base analog. For example, generosity of spirit may be realized through good-natured joking and the pleasantness of the object may be realized by some property (e.g., incongruence) of the joke to cause laughter. The analogy to the base only becomes apparent as the construal of the target in terms of a common relational category is realized.

The present goal is to build upon the findings of Minervino et al.’s (2013) that compared situations can become “analogies without commonalities” if both are canonical members of the same relational category. In the current experiments, we make one key shift to extend beyond existing work: instead of presenting two canonical cases of the relational category, we present pairs that consist of one canonical case and one target case where the latter is generally not seen as a member of the category on its own (but may be subject to re-categorization). Further, we now employ self-reported construal of the target case as the dependent measure (rather than analogical acceptance).

Analogy constitutes an instrument to detect that two initially mismatching relational predicates are similar (Gentner and Wolff, 2000), to highlight relational commonalities by creating a focus on a subset of relevant information (Kurtz et al., 2013) or to generate new information that can prove valid for the target (Blanchette and Dunbar, 2002). However, most of these re-representational mechanisms have been conceived as methods for discovering the intersection between base and target relations that were all there to begin with. In line with the idea that some metaphors cannot only discover but also create similarities (see, e.g., Indurkhya, 1992), in the present study we set forth to document an analogical mechanism capable of eliciting a novel perception of the target that cannot be conceived merely as resulting from drawing our attention to preexisting information in the target, but one that supposes an alteration of our initial conceptualization of the target. While in Experiment 1 the base-target pairs are explicitly introduced as constituting an analogy, in a follow-up study we presented the base-target pairs for analogical evaluation prior to eliciting a description of the target case. In this way we were able to assess whether the predicted effect emerges from natural comparison or whether it requires an explicit framing of the two cases as analogically related.

## Experiment 1

Participants in the analogy group read a canonical example of a schema-governed category followed by a non-canonical example of such category. Their task on each trial was to describe the situation of the target given that it was analogous to the base. Participants in the no-analogy group received the target case after reading a non-related situation and were asked to describe the target. The descriptions of the target case were analyzed to determine the extent to which participants in both groups applied the relational category corresponding to the base analog of the experimental condition.

### Methods

#### Participants

Sixty undergraduate students from the Comahue University participated in the experiment for course credit. Participants were randomly assigned to form even-sized groups experiencing the analogical condition and non-analogical conditions. All subjects gave written informed consent in accordance with the Declaration of Helsinki.

#### Procedure and Materials

Participants in the *analogical condition* read a definition of analogy accompanied by two examples (see Table 1). The general instruction for the task stated that they would receive several pairs of analogous situations, with the task of providing one description of the second situation of each pair. After reading this instruction, participants received a booklet containing six critical trials randomly interleaved with six filler trials. Each critical trial presented a situation that would normally be categorized as being an exemplar of certain schema-governed category (e.g., hanging garlic on the door as a case of *superstitious behavior*) followed by a second situation that – despite being a possible exemplar of that category – would not be spontaneously categorized as such (e.g., lighting a candle in the basement; the full set of critical materials is presented in Table 2).

**Table 1:** Instructions given to participants Experiment 1.

Analogical condition	Non-analogical condition
**Definition of analogy**	
“Two things are analogous when they are similar in essential respects but differ in superficial aspects”.	———-
**Examples provided**	
“Example 1: Peter kicked Susan’s ankle is analogous to Jeff pulled Tom’s hair.	
Example 2. Larry rented a house is analogous to Larry bought a yacht”	
**General instruction**	
“You will receive several pairs of analogous situations. For each pair, you will have to provide a description of the second situation”.	**General instruction**“You will receive several pairs of situations. For each pair, you will have to provide a description of the second situation”.
**Example of a critical trial:**	**Example of a critical trial:**
”Situation1: john gave a perfume to María.	”Situation 1: John lodged a complaint on María.
Situation 2: john played a joke on María.	Situation 2: john played a joke on María.
How would you describe the second situation considering that it is analogous to the first one?”	How would you describe the second situation?”


**Table 2:** Critical sets of materials Experiments 1 and 2.

Category	Exemplar type	Specific situation
Romantic courtship	Canonical	Juan gave a perfume to María (Juan le regaló un perfume a María)
	Non-canonical	Juan played a joke on María (Juan le hizo una broma a María)
	Non-analog	Juan lodged a complaint against María (Juan le presentó una demanda a María)
Marital infidelity	Canonical	Ariel closed the chat when his wife arrived (Ariel cerró el chat cuando entró su mujer)
	Non-canonical	Ariel made the bed when his wife arrived (Ariel arregló la cama cuando entró su mujer)
	Non-analog	Ariel blew his nose when his wife arrived (Ariel se sonó la nariz cuando entró su mujer)
Superstition	Canonical	Dolores hung garlic on the house door (Dolores colocó ajos en la puerta de la casa)
	Non-canonical	Dolores lit a candle in the basement (Dolores prendió una vela en el sótano)
	Non-analog	Dolores forgot a jacket in the backyard (Dolores se olvidó la campera en el jardín de su casa)
Cooling off	Canonical	Fernando took off his t-shirt after cycling several kilometers (Fernando se quitó la remera después de bicicletear unos kilómetros)
	Non-canonical	Fernando entered a mall after exiting the subway (Fernando entró en un shopping después de salir del subterráneo)
	Non-analog	Fernando stayed overtime after completing his shift (Fernando hizo horas extra después de cumplir su turno)
Job seeking	Canonical	Vanina sent her CV to a job agency (Vanina envió su curriculum a una consultora)
	Non-canonical	Vanina read the newspaper in the bar (Vanina leyó el diario en el bar)
	Non-analog	Vanina spent the whole afternoon seeking the keys (Vanina estuvo buscando las llaves toda la tarde)
Competition	Canonical	Marcos challenged his classmates about who got the best grades in the quiz (Marcos desafió a sus compañeros a ver quién tenía mejor nota en el parcial)
	Non-canonical	Marcos queried his colleagues about their cars before buying his own car (Marcos preguntó a sus colegas qué auto tenían antes de comprar el suyo)
	Non-analog	Marcos gave his son a large collection of cigarette packets (Marcos regaló a su hijo su amplia colección de paquetes de cigarrillos)


Beneath each pair of sentences a prompt was provided that asked participants to write a description of the second situation under the assumption that it was analogous to the first: “How would you describe the second situation considering that it is analogous to the first one?” The inclusion of filler sets served to discourage participants from inferring that all cases could be resolved by applying the categorization of the base analog to the target. Toward this end, the structure of the filler sets was the opposite of the critical ones: while the second situation was chosen to be spontaneously categorized in terms of the critical schema-governed category, the former one pertained to the category but would not be spontaneously categorized as an exemplar of it. All trials (critical or filler) involved different schema-governed categories.

The overall instruction for participants in the *non-analogical condition* stated that they would receive several pairs of situations, with the task of providing a description for the second situation of each pair. After reading this instruction, participants received a booklet containing the same 12 target analogs as in the analogical condition, but they were preceded by a non-exemplar of the critical category. Each base non-analog involved a different schema-governed category. Beneath each pair of sentences a prompt was provided that asked participants to write a description of the second situation: “How would you describe the second situation.” The order of presentation of the critical and filler trials was counterbalanced across conditions. The experiment was individually administrated by computer in an experimental session that lasted approximately 30 min.

A preliminary study was conducted in order to select appropriate materials. After constructing 33 candidate sets each comprised of a canonical, a marginal, and a non-exemplar of a schema-governed category, the 99 situations were presented in randomized order to an independent group of 27 participants who were asked to produce descriptions of each situation. We selected 12 sets of materials for which: (1) the canonical exemplars were cases for which more than 60% of participants used the critical category to describe the situation; (2) the marginal exemplars were cases in which less than 15% of participants used the critical category to describe the situation; and (3) the non-analogs were situations for which none of the participants applied the critical category. Accordingly, it was clearly established in advance that the target analog had a low probability of being initially represented as an example of the critical category of the base.

#### Data Analysis

Two independent judges received each participant’s descriptions of the target analogs coupled with its critical corresponding schema-governed category, and had to decide in which cases the description corresponded to such category. They were instructed to consider as hits only those cases in which the exact critical concept or a very close synonym was employed (e.g., *witchcraft* or *ritual* instead of *superstition*). Judges agreed in 86% of the cases, and solved cases of disagreement by discussion.

### Results and Discussion

On average, participants in the no-analogy group used the critical category in 5.7% of the trials. In contrast to this low proportion, participants in the analogical group used the critical category to refer to the target analogs in 52.14% of the trials. This difference between groups was significant, *M* = 0.5214, *SD* = 0.1993 vs. *M* = 0.057, *SD* = 0.0801, *t*(50.23) = 13.473, *p* < 0.001.

Despite the dramatic increase in the proportion of these hitherto unlikely categorizations of the target analogs, a limitation in the generalizability of this result is the fact that participants were asked to describe the second situation under the assumption that it was analogous to the first. Even though this external pressure to consider two situations as analogous is representative of the use of analogy in a myriad of everyday activities such as persuasion, explanation, or instruction, there are other circumstances in which a prompt to compare two situations does not explicitly favor the acceptance of their analogical relatedness. In order to assess whether the results of Experiment 1 generalize to less directive contexts, we introduced a variation that allowed for evaluation of a spontaneous analogical categorization instead of a forced one.

## Experiment 2

In this experiment we introduced the use of a yes-no question regarding the analogical relation between the two presented situations prior to the categorization task. That is, participants read two situations and were asked whether they regarded them as analogous. Following a within-subjects design, unlike Experiment 1, all participants were shown analogy and non-analogy item types with the number of expected “yes” and “no” answers balanced. After making the analogical acceptance judgment, participants were asked to describe the second situation. We assessed whether comparing the target to a base analog (in contrast to a base non-analog) would increase the proportion of cases in which the category that was naturally applicable to the base analog of the analogical condition was used to categorize the target situation.

### Methods

#### Participants

Thirty-six undergraduate students from the Comahue University participated in the experiment for course credit. All subjects gave written informed consent in accordance with the Declaration of Helsinki.

#### Procedure and Materials

Participants read a definition of analogy accompanied by an example of an analogy and an example of a non-analogy (see Table 3). They were informed that they would receive pairs of situations with the task of determining whether they were analogous or not (“Do you consider that these two situations are analogous?”). Following that task they were asked: “Then, how would you describe the second situation?”

**Table 3:** Instructions given to participants Experiment 2.

**Definition of analogy** ”Two things are analogous when they are similar in essential respects but differ in superficial aspects”.
**Examples:**
”Example of analogy: Peter kicked Susan’s ankle is analogous to Jeff pulled Tom’s hair.
Example of non-analogy: Larry rented a house is not analogous to Jill ate a candy”.
**General instruction**
”You will receive several pairs of situations. For each pair, you will begin by determining whether they are analogous or not. After this, you will be asked to describe the second situation”.
**Example of a critical trial:**
”Situation 1: John gave a perfume to María
Situation 2: John played a joke on María” (analogical condition)
or
”Situation 1: John lodged a complaint on María
Situation 2: John played a joke on María” (non-analogical condition)
Do you consider that these two situations are analogous? Yes–No.
Then, how would you describe the second situation?”


We used the same materials as in Experiment 1. As all participants were presented with analogies and non-analogies, we were able to implement a within-subjects manipulation of whether the critical target analogs were preceded by a canonical exemplar of the category to which they belonged. Each participant received three critical analogies, three critical non analogies, three filler analogies and three filler non analogies. The order of presentation of the sets was counterbalanced. For each participant who received the critical analogy version of Set 1 (for instance), another participant received the critical non-analogy version of the same set.

### Results and Discussion

Following the same criteria from Experiment 1, two independent judges evaluated whether participants used the critical category to describe the target analog. Judges agreed in 84% of the trials. Cases of disagreement were solved by discussion. On average, in the no-analogy condition participants used the critical category in 7.11% of the trials. Participants in the analogy condition accepted the analogy in 69.44% of the trials. Closely following the pattern seen in Experiment 1, participants who accepted the analogy used the critical category to refer to the target analogs in 57.17% of the trials. Once again this difference was significant, *M* = 0.5717, *SD* = 0.3464 vs. *M* = 0.0711, *SD* = 0.2029, *t*(34) = 8.134, *p* < 0.001.

Even though the majority of participants who answered “yes” to the question of analogical relatedness invoked the critical category, a non-negligible proportion of participants did not invoke the critical category for characterizing the target situation. An informal analysis of these descriptions showed that while in 13.81% of the cases participants seemed to have applied the target category to the base (e.g., they categorized the target *playing a joke* as a case of *wanting to gladden her heart* – a description that seems also applicable to the base case of *giving a perfume*), in 29.05% of the cases participants invoked a description that encompassed both facts (e.g., “wanting to be friendly,” which seems to be applicable to both giving a perfume and playing a joke).

The results of Experiment 2 accord in a convincing manner with those of Experiment 1. Given that in this second experiment participants had the freedom to determine if the two situations were analogous, these results allow us to dismiss the hypothesis that the results obtained in our first experiment were due to pressure exerted on participants to consider the two situations as analogous.

## General Discussion

Most theories of analogical mapping accept that two situations can be considered analogous even when their corresponding relations are not initially represented as having identical meaning (Gentner and Kurtz, 2006). They require, however, that non-identical relations can be re-represented in a way that at least some common semantic aspects of the relations become manifested. Standard re-representation mechanisms such as superordination (Falkenhainer, 1990) and decomposition (Yan et al., 2003) require that the meanings of the compared relations remain unmodified when these relations are bound to their respective arguments. To illustrate, as the meaning of *rent* and *borrow* are preserved when applied, respectively, to *house* and *van*, the preexisting, context-free similarity between these relations is preserved.

We contended that although the standard approach to re-representation can successfully cope with analogies in which the nucleus of semantics lies at relations, it might fail to capture alternative re-representation mechanisms that operate when comparing propositional structures in which there is a strong interdependency between the fillers that might instantiate the different thematic roles of such structures. In the present study we obtained evidence for a re-representation mechanism that consists in projecting a schema-governed category (Markman and Stilwell, 2001) from a canonical to a non-canonical exemplar of such category.

It could be argued that the analogies included in our materials do not require re-representation mechanisms any different from those traditionally proposed. As the meaning of verbs can be significantly altered as a function of the particular arguments to which they are bound (Gentner and France, 1988; Kersten and Earles, 2004), the concepts used as arguments in our base and target relations could have activatated particular connotations of such verbs, which happened to match (a contrapositive version of this kind of hypothesis was posited by Gentner and Kurtz (2006), to account for some anomalous cases in which participants denied analogical relatedness to pairs of situations whose relations maintained a clear semantic relatedness when considered in isolation). However, this explanation does not seem to hold for our materials, since the entities to which the verbs are applied neither activate an alternative meaning of the verbs themselves, nor do they promote an extended (e.g., metaphorical) use of those verbs. Rather, they activate concepts whose meaning are entirely different from the verbs on which they originate. Taking as an example the analogy between *hanging garlic from the door* and *lighting a candle in the basement*, the idea of *superstition* is neither contained in the base relation *hanging*, nor in the target relation *lighting*. Hence, the re-representation processes that were at work during the processing of our target situations do not reduce to highlighting a marginal semantic component common to the base and target relations.

Dominant accounts of analogical mapping agree in allowing the re-representation of two non-identical relations that are concurrently active in working memory. However, there is some debate as to whether candidate situations that are stored in long-term memory (LTM) could be re-represented to match a target situation currently active in working memory (Dietrich, 2000). While retrieval algorithms that rely on the identicality of base and target predicates (e.g., MAC/FAC, Forbus et al., 1995) allow re-representation of base situations once they have been retrieved, the algorithms proposed by the multiconstraint theory can probe LTM for situations containing non-identical predicates either by means of minimal ascension along external networks such as WordNet (ARCS, Thagard et al., 1990) or else by decomposing predicates and entities into their semantic primitives (LISA, Hummel and Holyoak, 1997). The results of the present study suggest yet another way in which re-representation may subserve analogical retreival, namely, by means of seeking further exemplars of the schema-governed category to which the target analog belongs. Upon a failure to evoke exemplars of the target category of events (e.g., prior flitatious behavior of person X toward person Y), a sensible retrieval strategy could consist in (1) breaking down the relational structure of such category into separable subcomponents (e.g., Person X’s manifestations of interest in Person Y), (2) probing LTM for instances of such component alone (e.g., Person X having played a joke on Person Y), and (3) assessing whether the retrieved episode can be ascribed to the “flirtatious behavior” relational category.

The present study has focused on how a dubious exemplar of a schema-governed category can be categorized as belonging to such category by way of comparing it to a more representative exemplar. Given that exemplars of a schema-governed category may display different values along critical dimensions of the category at stake (Tavernini et al., 2017), a future line of research could delve into whether an exemplar scoring low on a given dimension of such category could be re-represented as a result of being compared with an exemplar displaying higher values in such dimension (e.g., analogizing between a multimillion robbery a smaller one). Moreover, this kind of base situation need not consist in real episodes. For those target situations for which we cannot identify a known situation scoring higher (or lower) on the particular dimension that we want to emphasize, the *ad hoc* fabrication of a suitable base analog seems a sensible way to go. A naturalistic example of analogy fabrication in the service of re-representation was reported by Tony Veale during Analogy09: At a time when there was debate around whether public spaces should have a separate sector for smokers vs. prohibit smoking altogether, a bar displayed a sign which stated that “having a smoking sector at a pub is like having a peeing sector at a swimming pool” (Veale, 2009). The analogy promotes the re-representation of the target situation by highlighting a set of features that are more easily perceivable in the invented scenario.

A relevant question raised by the present results concerns the duration of the re-representation effects that may arise from the recategorization mechanism proposed herein. Going back to the initial example of the paper, the duration of a re-representation such as that experienced by the protagonist of “Soccer or me” could span from lasting just a moment to exerting a long-lasting effect on his behavior. Eventually, a fraction of these re-representations might end up changing the way in which the target phenomenon gets socially represented, giving birth to a lexicalized new concept like “soccerholism”. As proposed by Hofstadter and Sander (2013), not only does our culture provide us with potent concepts; it also encourages us to analogically extend them, giving rise to completely new families of concepts (*workaholism, chocoholism*, *pornoholism, technoholism*, etc.). On occasions, however, the re-representation of new events in terms of a schema-governed category will depend not only on cold cognitive insights but also on breaking cultural resistances. To illustrate, the recognition of sexism, ageism, speciesism or weightism as forms of discrimination took much more than just acknowledging a shared semantic structure across these concepts.

Despite its theoretical importance, the phenomenon of analogical re-representation has been the subject of very little empirical work. The present study contributes to documenting the existence of this rather elusive phenomenon, and opens new avenues for inquiry that depart from those traditionally proposed in the literature.

## Data Availability

The raw data supporting the conclusions of this manuscript will be made available by the authors, without undue reservation, to any qualified researcher.

## Ethics Statement

An ethics approval was not required for the present research as per the National Council for Scientific and Technical Research’s (CONICET) guidelines and Argentinian regulations.

## Author Contributions

NO designed the studies, administered the experiments, carried out the data analysis and participated in the literature review and the writing of the manuscript. MT collaborated in the data analysis and the writing of the manuscript. KK participated in the theoretical framing of the study and in the writing of the manuscript. RM participated in the design of the studies and the writing of the paper.

## Conflict of Interest Statement

The authors declare that the research was conducted in the absence of any commercial or financial relationships that could be construed as a potential conflict of interest.
